# Putting Knowledge into Practice—The Challenge of Acquiring Healthy Habits during Pregnancy

**DOI:** 10.1055/s-0039-1692633

**Published:** 2019-06-27

**Authors:** Ana Carolina Godoy-Miranda, Jessica Fernandes Cirelli, Maira Pinho-Pompeu, Daiane Sofia Morais Paulino, Sirlei Siani Morais, Fernanda Garanhani Surita

**Affiliations:** 1 Department of Obstetrics and Gynecology, Universidade Estadual de Campinas, Campinas, São Paulo, SP, Brazil

**Keywords:** healthy habits, gestation, weight gain, nutrition, physical exercise, antenatal education, hábitos saudáveis, gestação, ganho de peso, nutrição, exercício físico, educação pré-natal

## Abstract

**Objective** The aim of this study was to investigate the knowledge concerning gestational weight gain (GWG), nutrition, and physical exercise (PE) in pregnant women, and how to put them into practice.

**Methods** A cross-sectional study with 61 pregnant women above 26 weeks of gestation, at the Woman's Hospital, CAISM, University of Campinas. Questionnaires regarding the knowledge of healthy habits (HH) during pregnancy, sociodemographic data, and previous obstetric outcomes were applied. An educational guide with advice on HH during pregnancy and in the postpartum period was offered.

**Results** The average age of women was 28.7 ± 6.23 years, with 85% of them being married; 32% nulliparous; the average body mass index (BMI) before pregnancy was 25.4 ± 9.8 kg/m^2^, and the mean number of years of schooling was 11.2 ± 3.8. Only 61% of the subjects had received any previous information about GWG during their antenatal care and were aware as to how many pounds they should gain during pregnancy. Among the 61 women, 85% understood that they did not need to “eat for 2” and 99% knew that PE had benefits for their body and was safe for their baby. Half of the women practiced PE prior to pregnancy; however, only 31% continued the practice of PE during the pregnancy.

**Conclusion** Despite understanding the need for HH during pregnancy, women still need encouragement to practice PE during pregnancy, as well as more information about GWG.

## Abstract (pt)

**Resumo:**

**Objetivo** O objetivo deste estudo foi investigar os conhecimentos sobre ganho de peso gestacional (GPG), nutrição, e exercício físico (EF) em gestantes e o quanto elas os colocam em prática.

**Métodos** Estudo transversal realizado no Hospital da Mulher, CAISM, Unicamp, com 61 gestantes acima das 26 semanas gestacionais. Questionários sobre conhecimento de hábitos saudáveis (HS) durante a gestação, dados sociodemográficos, e antecedentes obstétricos foram aplicados. Um guia educacional com conselhos sobre HS durante a gravidez e período pós-parto foi oferecido.

**Resultados** A idade média das mulheres foi de 28,7 ± 6,23 anos, sendo 85% casadas, 32% nulíparas, o índice de massa corporal (IMC) médio antes da gestação foi de 25,4 ± 9,8 kg/m^2^, e a média de anos de escolaridade foi de 11,2 ± 3,8. Apenas 61% das mulheres entrevistadas haviam recebido informações prévias sobre o GPG durante o pré-natal e sabiam quantos quilos deveriam ganhar durante a gravidez. Entre as mulheres, 85% sabiam que não precisavam “comer por dois,” e 99% sabiam que o EF tinha benefícios para seu corpo e era seguro para seu bebê. Metade das mulheres praticava EF antes da gravidez, mas apenas 31% continuaram praticando durante a gravidez.

**Conclusão** Apesar de compreender a necessidade de HS durante a gravidez, as mulheres ainda precisam de incentivo para praticar EF durante a gravidez, bem como mais informações sobre o GPG.

## Introduction

Adopting healthy habits (HHs) during pregnancy is essential for adequate fetal development and maternal well-being. Knowledge regarding gestational weight gain (GWG) is primordial if women are to present adequate weight gain during pregnancy. However, most antenatal physicians and healthcare providers do not have appropriate training to advise pregnant women on adequate weight gain.^1^

Physical exercise (PE) is an important tool to control weight gain during pregnancy and has been gaining increasing popularity among women of childbearing age.^2^ ^3^ Physical exercise is considered a safe practice, provided that frequency, intensity, and duration are respected. In the absence of absolute contraindications, women should be encouraged to engage in regular PE.^4^ ^5^ The prevalence of physically active pregnant women, as well as the duration, frequency, and intensity of exercise are lower than those of adult women in general.^2^ ^5^

Knowledge of the practice of PE during pregnancy by pregnant women is also extremely important. In a study performed in women treated by the united healthcare system in Brazil, 25% of the women were sufficiently informed about the practice of PE in pregnancy, while only 18% performed PE according to recommendations, with “lack of time” (55.8%) reported as being the primary cause.^6^ Similarly, in a study of 61 pregnant women, 31.1% of them reported performing PE at a moderate intensity (150 minutes per week), with “fatigue” being the most frequent cause for pregnant women not practicing PE.^7^

Food quality and nutritional status play an important role in GWG, affecting fetal growth and development, as well as the future woman's weight. Differentiated nutritional care in this period is essential, particularly for pregnant women at weight extremes.^8^

Nutritional status, diet and PE are modifiable risk factors that can be altered through effective interventions before and during antenatal care. Therefore, implementing knowledge about healthy lifestyle habits during pregnancy is the first step in promoting PE and appropriate diet, thus maintaining an acceptable weight during this period.^9^ The aim of the present study was to evaluate pregnant women's understanding of HHs and the practice of PE during pregnancy.

## Methods

The present study was approved by the Research Ethics Committee of Universidade Estadual de Campinas (CAAE: 4.6903115.3.0000.5404), and all items of the Strengthening the Reporting of Observational Studies in Epidemiology were followed. A cross-sectional study was designed. Data collection was performed at the Antenatal Outpatient Clinic of Women's Health, at Universidade Estadual de Campinas, Campinas, SP, Brazil, a public university hospital, from October 2015 to January 2016. Women with gestational age ≥ 26weeks, singleton pregnancy, over 18 years of age, without contraindications to practice PE during pregnancy, who were undergoing antenatal care at the aforementioned institution, were invited to participate in this study. Gestational age was not an inclusion criterion, and pregnant women at all gestational ages were invited to aid in this study. Women with reading or communication difficulties and those with any condition that may interfere with the understanding of the questions were excluded. After agreeing to participate in the study, an informed consent form was signed.

The participants replied to questions regarding sociodemographic data (age, family income, schooling, paid work, marital status, race), obstetric history (planned pregnancy, parity miscarriage, prepregnancy weight), and a question about practice of PE before and during pregnancy. They also answered a questionnaire on the knowledge of HHs during pregnancy, regarding GWG, nutritional recommendations, and PE throughout pregnancy (Chart 1).

**Chart 1:** Questions about knowledge of healthy habits during pregnancy that formed the knowledge score with correct answers between parentheses

1. Do you think it is important to know how much you weighed before you became pregnant? (YES) 2. Do you think prepregnancy weight interferes with a woman's pregnancy? (YES) 3. Do you know how much weight you should gain by the end of your pregnancy? If so, how much do you think you can gain until the end of your pregnancy?* 4. Do you think that you should “eat for two” during pregnancy? (NO) 5. How many liters of water do you think you should drink per day during pregnancy?** 6. Can consuming large amounts of coffee, soda, Coca-Cola and matte tea be harmful to a baby's health? (YES) 7. Which of the foods below should be consumed daily? (FRUIT AND VEGETABLES, GRILLED MEAT, MILK AND DERIVATIVES WITH LOW FAT)*** 8. Does the consumption of meats, dark leafy greens, and beans help prevent anemia during pregnancy? (YES) 9. Symptoms like nausea, vomiting, and heartburn are very common during pregnancy. Does eating small meals every 3 hours help to improve these symptoms? (YES) 10. Have you heard about exercise for pregnant women? (YES) 11. In your opinion, is physical exercise during pregnancy necessary? (YES) 12. Do you think exercising during pregnancy has any benefits for your body? (YES) 13. Do you think exercise is safe for your baby? (YES) 14. Is creating healthy habits during pregnancy important to you? (YES) 15. Do you think that creating healthy habits during pregnancy is important for your baby? (YES)

* The values of gestational weight gain according to the classification of prepregnancy body mass index for each pregnant woman were considered correct: Low weight = 12.5 to 18 kg; Eutrophic = 11.5 to 16 kg; Overweight = 7.0 to 11.5 kg; Obesity = 5.0 to 9.0 kg (IOM, 2009).

** Values between 1.5 and 2.5 L of water per day were considered correct.

*** Answers with only one or two food groups were considered incorrect.

The interviews were face-to-face and with a mean duration of 30 to 40 minutes. They place during the waiting for medical attention in the waiting room, avoiding damage in the prenatal care routine. All interviews were conducted by high school students, who were trained and supervised by the head investigator.

Following the interview, the pregnant women received an educational guide containing recommendations for HHs during pregnancy and in the postnatal period, elaborated and developed by the research team, aimed at stimulating pregnant women to adopt an adequate diet, follow PE regimens and understand appropriate GWG.

Woman's height, weight and gestational age at the first prenatal care visit, weight at the end of prenatal care, GWG, newborn weight and gestational age at birth were collected from medical records and through an online hospital intranet CAISM system, (Campinas, SP, Brazil). Gestational weight gain was classified based on the categories of prepregnancy BMI, according to the recommendations of the Institute of Medicine, and then classified into the following categories: insufficient, adequate or excessive.^10^

Calculation of sample size using the proportion of women who practice PE during pregnancy (65.6%) was considered to estimate the proportion of women who will present a good knowledge.^6^ A significance level of 5% and a sample error of 15% (prevalence estimated between 51.6 and 79.6%) were used, and the resulting sample size was 46. However, due to possible losses during data collection, 61 women were included in this study.

To calculate the understanding of healthy habits, a score was created based on 15 questions that addressed the knowledge of pregnant women about GWG, healthy eating and the practice of PE during pregnancy (Table 1). Each correct response corresponded to 1 point, while each incorrect reply received 0 points, therefore allowing a score between 0 (minimal knowledge) and 15 (maximum knowledge). From the total number of correct answers, the women were classified as having excellent knowledge of healthy habits (> 11 points), good knowledge of healthy habits (7–11 questions) and poor knowledge of healthy habits (< 7 questions).

**Table 1:** Obstetric and sociodemographic characteristics of pregnant women

**Variables**	**Mean ± SD**
Schooling (years completed)	11.2 ± 3.8
Family income (R$)	2,587.1 ± 1,705.3
Gestational age at 1st PN visit (weeks)	13.8 ± 6.1
BMI prepregnancy (Kg/m^2^)	27.5 ± 9.8
Gestational weight gain (kg)	12.1 ± 13.3
**Variables**	**n (%)**
Race	
Caucasian	29 (48.3)
Other	31 (51.7)
Missing	1
Marital status	
Stable union	51 (85.0)
Without stable union	9 (15.0)
Missing	1
Employed during pregnancy	35 (58.3)
Parity	
Primiparous	27 (44.3)
Multiparous	34 (55.7)
Planned pregnancy	33 (54.1)
Hypertensive syndromes	5 (8.2)
Diabetes Mellitus	5 (8.2)
BMI categories prepregnancy	
Low weight	4 (6.7)
Normal	23 (38.3)
Overweight	13 (21.7)
Obese	20 (33.3)
Missing	1

Abbreviations: BMI, body mass index; PN, prenatal; SD, standard deviation.

### Statistical Analysis

Descriptive variables were studied through mean, standard deviation and frequency. As the questionnaire about knowledge of HHs and PE during pregnancy is not validated, the authors present at least an analysis of internal consistency of Cronbach's α. The association between the knowledge score and the GWG was assessed using the Spearman correlation index. The difference between the variables according to the knowledge category was evaluated through the Mann-Whitney test. Knowledge of HHs was evaluated according to the prepregnancy body mass index (BMI) using the Mann-Whitney test. The level of significance considered was 5%, using the SAS version 9.4 software (SAS Institute Inc., Cary, NC, USA).

## Results

A total of 106 pregnant women were invited to participate in this study, 13 (12.3%) of whom refused to participate in the study, and 32 (30.2%) who presented a relative contraindication to the practice of PE. Therefore, a total of 61 pregnant women were included, with a mean age of 28.7 ± 6.2 years. About 55% of the women studied were classified as being above the ideal weight, according to their BMI (obese or overweight), and GWG was adequate in only 23% of them. Others obstetrics and sociodemographic characteristics are described in (Table 1).

The mean level of knowledge of HHs was 12.7 ± 1.8, ranging from 8 to 15 points. No pregnant woman scored < 7, considered low knowledge of healthy habits (data not shown). The results of the knowledge questionnaire were evaluated by internal consistency of Cronbach's α = 0.54.

The present study also demonstrated that only 29.5% of the women were aware of GWG, and 45 (73.8%) were evaluated for GWG up to and including the end of pregnancy. The mean GWG was 12.1 ± 13.3 Kg, and according to the prepregnancy BMI, the GWG was adequate in only 23% of the women. All women considered underweight according to their BMI presented adequate weight gain, while among those classified as eutrophic, overweight, or obese, more than half had excessive GWG. More specifically, among the women classified as overweight, 22.3% were observed to have insufficient, 11.1% adequate and 66.6% excessive GWG. In obese women, these numbers were 18.2%, 27.2% and 54.6%, respectively (data not shown).

The sources and types of information received by the pregnant women on HHs during pregnancy were grouped as shown in Table 2. Most women also reported having heard of PE during pregnancy (87%), with 49% practicing PE prior to pregnancy and only 31.1% continuing this habit during the antenatal period.

**Table 2:** Sources of information received regarding healthy habits and practice of physical exercise according to body mass index and level of knowledge of healthy habits (*n* = 61)

Questions	n	%
Were you questioned about gestational weight gain during the prenatal period? (*n* = 60)		
Yes	40	66.7
No	20	33.3
Which professional questioned you about gestational weight gain? (*n* = 40)*		
Doctor	29	72.5
Nurse	8	20.0
Nutritionist	3	7.5
Were you advised about gestational weight gain during the prenatal period?		
Yes	18	29.5
No	43	70.4
Who offered you information about gestational weight gain? (*n* = 18)*		
Doctor	15	83.3
Nurse	2	11.1
Nutritionist	1	5.5
What type of information did you receive? (*n* = 18)*		
Dietary advice	11	73.3
Weight gain limits (up to 10 kg or 20 kg)	4	26.7
Have you heard of physical exercise during pregnancy?		
Yes	53	86.9
No	8	13.1
How did you find out about physical exercise during pregnancy? (*n* = 53)*		
Internet	19	35.8
Doctor	11	20.7
Television	10	18.8
Books and magazines	5	9.4
Others	7	13.2

* These questions were asked only if the answer of the previous question was “Yes”.

The non-practice of PE before pregnancy was observed in 50% (30) of women, 32.7% (20) practiced walking, and 16.3% (10) practiced another type of PE. Among women who practiced PE before gestation, 46.7% (14) practiced it more than 5 time per week. During pregnancy, 67.8% (40) of the women did not practice PE, 25.4% (15) practiced walking and 6.8% (4) practiced another time of PE. Among the women who practices PE during pregnancy, 21% (4), practiced it more than 5 times per week. There were no significant associations between PE during pregnancy, prepregnancy BMI and level of knowledge of HHs (Table 3).

**Table 3:** Association between practicing physical exercise during pregnancy with prepregnancy body mass index and the level of knowledge about healthy habits

	Practiced PE (*n* = 19)		Never Practiced PE (*n* = 39)	
Prepregnancy BMI	n	%	n	%
Low weight	2	10.5	2	5.1
Normal	9	47.4	15	35.9
Overweight	3	15.8	9	23.1
Obesity	5	26.3	14	35.9
**Knowledge level**				
Good	4	21.1	9	22.5
Excellent	15	78.9	31	77.5

Abbreviations: BMI, body mass index; PE, physical exercise.

Pregnant women with “excellent” knowledge of HHs presented higher average income, lower parity, and number of births, and lower GWG, when compared with pregnant women with “good” knowledge (*p* = 0.0213, 0.0405, 0.0484 and 0.0317, respectively) (Table 4).

**Table 4:** Evaluation of sociodemographic characteristics, obstetric history, and gestational weight gain according to the knowledge of healthy habits during pregnancy (*n* = 61)

	Good knowledge (7–11 points)			Excellent knowledge (> 11 points)			
	n	Median	Mean ± SD	n	Median	Mean ± SD	*P*-value
Age (years)	14	29.5	30.0 ± 6.8	47	27.0	28.3 ± 6.1	0.3663
Income (R$)	12	1,150.0	1,710.0 ± 1,087.7	45	2,000.0	2,821.1 ± 1,771.9	0.0213
Schooling (years)	13	11.0	10.2 ± 5.2	46	11.0	11.5 ± 3.4	0.9631
Parity	14	3.0	3.4 ± 1.6	47	2.0	2.6 ± 2.1	0.0405
Miscarriages	14	0.50	0.79 ± 1.05	47	0.00	0.70 ± 1.53	0.2767
Births	14	1.50	1.86 ± 1.70	47	1.00	0.87 ± 1.10	0.0484
Prepregnancy weight (Kg)	13	59.0	71.1 ± 31.3	47	67.0	73.0 ± 22.4	0.3684
Weight at the start of PN (Kg)	14	63.1	70.1 ± 17.7	42	68.4	73.5 ± 20.7	0.4751
Weight at end of PN (Kg)	12	86.5	80.6 ± 16.7	34	78.3	82.6 ± 19.0	0.9109
GWG (Kg)	12	16.7	21.6 ± 15.2	33	12.4	10.2 ± 10.4	0.0151
Newborn weight (g)	10	3,002.5	2,753.5 ± 1,020.6	31	3,360.0	3,262.6 ± 480.0	0.2317
GA at 1st PN visit	14	12.5	13.4 ± 6.0	39	13.0	14.3 ± 6.8	0.6872

Abbreviations: GA, gestational age; GWG, gestational weight gain; PN, prenatal.

There was a significant correlation between parity and the knowledge score (*p* = 0.0075 r = - 0.3393), and a borderline correlation between the number of births and the knowledge score (*p* = 0.0575 r = - 0.2445). These correlations were negative and weak, indicating that the higher the parity, the lower the knowledge scores. However, although the correlations were significant, the magnitude of this relationship was undetectable as the correlation was weak (r < 0.5).

## Discussion

The main findings of the present study were the high level of knowledge of pregnant women regarding the HHs that should be performed during pregnancy (score of 12.7 ± 1.8) and the low prevalence of PE in those who understand its benefit. The women with better HHs knowledge scores during pregnancy presented lower GWG, higher family income, lower parity, and lower number of births.

Access to information is widening, enabling women to have guidelines on HHs through social media, internet, newspapers, magazines, television, family, friends, educational activities on childbirth, and discussions with healthcare professionals.^11^ An Australian study showed that discussions with midwives were the main source of information used by women during pregnancy (70%), followed by the use of the internet (44%), different from this study, in which women ended up receiving information through social media before a health professional.^12^

A study of 166 women showed that knowledge about GWG is poor, with the minimum amount of weight gain during pregnancy being overestimated by most women, therefore positively promoting excessive weight gain during pregnancy.^13^ ^14^ Despite the complications related to excessive GWG being well described, few studies have evaluated the knowledge of pregnant women regarding the guidelines used to direct GWG.^15^ ^16^

In addition, we have found different GWG guidelines in the literature, which can be a confounding factor. A survey of mothers in the first year postpartum revealed that they have a poor understanding of these recommendations.^17^ ^18^ Another study^19^ considering prepregnancy maternal weight as a strong influence on the GWG, suggested that prepregnancy BMI is related to the accuracy of GWG knowledge. Most participants, regardless of their BMI, did not accurately estimate the minimum or maximum amount of weight that they could gain, with eutrophic, overweight, and obese women being more likely to overestimate the maximum amount of weight they should gain.^20^ In a study performed in 1,052 pregnant women in Campinas, higher rates of overweight (24.6%) and obesity (13.6%) were encountered. In addition, overweight (55.9%) and obese (53.7%) women have demonstrated the highest weight gain.^21^ ^22^

The lack of knowledge about current GWG recommendations, and the risks of excessive GWG has also been described in a study of 15 women with over 10 kg weight retention postpartum. In addition, questions about their weight during antenatal and postpartum care by medical professionals were scarce or absent.^21^

Regarding the practice of PE in pregnancy, just over half of the women in our sample had received some form of guidance, with 35.8% obtaining this information from the internet, and only 20.7% formally from their physician. Most of the pregnant women (86.9%) were aware that PE should be continued during pregnancy; however, of the 49% who practiced PE prior to pregnancy, only 31.1% continued to do it during the pregnancy.

A study of 161 women found that 65% were informed about the practice of PE, with most being in favor of it. However, as in the current study, only 20% of them exercised sufficiently.^23^ These results suggest that women's knowledge of PE is reasonable, with favorable attitudes; however, few actually exercise during pregnancy, leaving a gap in the literature as to the real reasons that cause pregnant women to not adhere to PE during pregnancy.^23^

To understand the breach between knowledge and practice of HHs during pregnancy, some authors have stated that pregnant women perceive their bodies in a fragmented way, part “pregnant” and part “woman.” On the other hand, they demonstrate confidence with their knowledge of HHs, yet, they do not always understand HHs during pregnancy, causing insecurities about how much food and PE can positively or negatively influence their pregnancy. Another factor that may influence GWG is the fact that they consider weight gain as acceptable, even when excessive, in terms of their pregnancy, around the abdominal region.^24^ Numerous interventional approaches to healthy lifestyle habits, have been designed over recent years. It is paramount that strategies taken during the antenatal period go beyond routine obstetric monitoring, aiming at promoting the physical and mental health of women in an integrated fashion; after all, pregnancy is an opportune moment to implement HHs. It is essential to identify strategies that reach out to this population in a more holistic way, integrating knowledge, body and psyche to guide and achieve women's adherence to an increasingly healthy lifestyle. An informed and participative pregnant woman during antenatal care becomes a collaborative partner for the healthcare team, as she acquires knowledge, skills, and confidence, which can provide several benefits, such as HHs. Engaging women as active participants in their healthcare improves treatment outcomes, in addition to greater satisfaction.^9^

The main limitation of this study is its small sample size in a specific region of Campinas, SP, Brazil. In addition, a non-validated questionnaire was used, exclusively designed for this research; therefore, this was the first time it was ever used. Even with all the interviewed women being ≥ 26 weeks of gestational age, the fact that women were interviewed at different gestational ages can also be considered a study imitation, since the timing of the interview could influence the HHs knowledge.

Although the questionnaire's internal consistency of Cronbach's α found was reasonable (0.54), since the ideal would be above 0.7, we emphasize that all the questions considered in the score had a positive correlation. The sample, though small, may be considered a pilot of the pregnant womens' knowledge about HHs, and, based on these data, the score should be validated with a larger sample.

## Conclusion

Most pregnant women know the importance of HHs during pregnancy; however, they do not know how to put this knowledge in practice. Antenatal education about PE, healthy diet and GWG can stimulate better contact between the pregnant woman and her healthcare team, therefore providing information and discussion of doubts that the pregnant woman may present, and encouraging these women to change her life habits. Antenatal education aims at empowering women, and demystifying negative aspects of pregnancy, such as “eating for two” and “resting,” facilitating changes in life habits and preventing negative perinatal outcomes. Additionally, pregnancy is a moment of opportunity to change unwelcome life habits for all life.
